# Low prevalence of ideal cardiovascular health in the general Swedish population: Results from the Swedish CArdioPulmonary bioImage Study (SCAPIS)

**DOI:** 10.1177/14034948221147093

**Published:** 2023-01-16

**Authors:** Sara Higueras-Fresnillo, Ángel Herraiz-Adillo, Viktor H. Ahlqvist, Daniel Berglind, Cecilia Lenander, Bledar Daka, Maria Brännholm Syrjälä, Johan Sundström, Carl Johan Östgren, Karin Rådholm, Pontus Henriksson

**Affiliations:** 1 Department of Health, Medicine and Caring Sciences, Linköping University, Sweden; 2 Department of Global Public Health, Karolinska Institutet, Sweden; 3 Centre for Epidemiology and Community Medicine, Region Stockholm, Sweden; 4 Department for Clinical Sciences in Malmö, Centre for Primary Health Care Research, Lund University, Sweden; 5 School of public health and community medicine, Sahlgrenska Academy, University of Gothenburg, Sweden; 6 Department of Public Health and Clinical Medicine, Family Medicine, Umeå University, Sweden; 7 Department of Medical Sciences, Uppsala University, Sweden; 8 The George Institute for Global Health, University of New South Wales, Australia; 9 Centre for Medical Image Science and Visualization (CMIV), Linköping University, Sweden

**Keywords:** Cardiovascular diseases, cardiovascular health, epidemiology, public health, risk factor

## Abstract

The aim of the current study was to examine the prevalence of ideal cardiovascular health (iCVH) in the general Swedish middle-aged population. To address this aim, we utilised data from the Swedish CArdioPulmonary bioImage Study (SCAPIS) which is a large Swedish population-based study (*N*=30,154) that combined comprehensive state-of-the-art imaging technology with clinical examinations and included all iCVH components. A total iCVH score was calculated as the number of iCVH metrics at an ideal level for the seven components and classified as: ideal (⩾5 ideal components), intermediate (3–4 ideal components) and poor (⩽2 ideal components). Our results showed that only 18.2% of the population reached ideal status (i.e. ⩾5 components at the ideal level), whereas 51.9% were classified as intermediate status and 29.9% as poor status of iCVH. Women had a higher prevalence of iCVH status (23.9% vs. 12.0%) and a lower prevalence of poor iCVH status (23.5% vs. 36.8%). Our data may serve as benchmarks for future national and international comparisons and motivate efforts to promote cardiovascular health in the general population, given the strong link between iCVH with all-cause and cardiovascular disease mortality and morbidity.

## Introduction

Cardiovascular diseases (CVD) are leading causes of morbidity and mortality globally [1,2]. To better monitor and improve cardiovascular health, the American Heart Association created the ‘ideal cardiovascular health’ (iCVH) construct [3]. This construct is based on three health factors (ideal levels of blood pressure, blood glucose and total cholesterol) and four health behaviours (non-smoking status, ideal body mass index (BMI), healthy diet and meeting physical activity recommendations). In the literature, there is evidence of a strong inverse association between the number of cardiovascular health metrics at the ideal level with all-cause and cardiovascular mortality, as well as incident cardiovascular diseases, disability and morbidity [4,5]. iCVH also provides the opportunity to analyse the prevalence and trends of the cardiovascular health of a population. Although the prevalence of iCVH has been reported in several populations globally [6], there are no contemporary data describing iCVH in a Scandinavian country.

Such information is important to tailor public health strategies to promote cardiovascular health. The aim of the current study was therefore to examine the prevalence of iCVH in the general Swedish middle-aged population. We also aimed to examine differences in iCVH according to sex. To address these aims, we utilised data from the Swedish CArdioPulmonary bioImage Study (SCAPIS) which is a large Swedish population-based study (*N*=30,154) that combined comprehensive state-of-the-art imaging technology with clinical examinations and included all iCVH components [7,8].

## Methods

SCAPIS randomly invited individuals from the census register at six cities in Sweden (Gothenburg, Linköping, Malmö/Lund, Stockholm, Umeå and Uppsala) between 2013 and 2018, and a total of 30,154 participants were included (participation rate: 49.5%). Physical examinations were performed on two or three occasions within a period of two weeks, and detailed information regarding the study methods has been reported elsewhere [7]. This study was approved by the Swedish Ethical Review Authority (2021-06408-01), and written informed consent was obtained from study participants.

SCAPIS included a total of 30,154 participants. All participants had data on at least one iCVH variable and were included in the current study. Definitions of the seven iCVH components and criteria for ideal, intermediate and poor levels are presented in Table I. A total iCVH score was calculated as the number of iCVH metrics at an ideal level for the seven components and classified as: ideal (⩾5 ideal components), intermediate (3–4 ideal components) and poor (⩽2 ideal components).

**Table I.** Definitions of ideal, intermediate and poor cardiovascular health for each metric.

Metrics	Definitions
*Behaviours*	
Smoking status	Ideal: Never smoker or quit >12 months ago Intermediate: Former smoker ⩽12 months ago Poor: Current smoker
Body mass index	Ideal: <25.0 kg/m^2^ Intermediate: 25.0–29.9 kg/m^2^ Poor: ⩾30.0 kg/m^2^
Physical activity	Ideal: ⩾150 minutes per week of moderate intensity or ⩾75 minutes per week of vigorous intensity or ⩾150 minutes per week of moderate-vigorous intensity Intermediate: 1–149 minutes per week of moderate intensity or 1–74 minutes per week of vigorous intensity or 1–149 minutes per week of moderate-vigorous intensity Poor: None
Diet	Ideal: 4–5 components at the ideal level^a^ Intermediate: 2–3 components at the ideal level^a^ Poor: 0–1 component at the ideal level^a^
*Factors*	
Total cholesterol	Ideal: <5.2 mmol/L (US: <200 mg/dL) untreated Intermediate: 5.2–6.1 mmol/L (US: 200–239 mg/dL) or treated to goal Poor: ⩾6.2 mmol/L (US: ⩾240 mg/dL)
Blood pressure	Ideal: SBP <120 mmHg and DBP <80 mmHg untreated Intermediate: SBP 120–139 mmHg or DBP 80–89 mmHg or treated to goal Poor: SBP ⩾140 mmHg or DBP ⩾90 mmHg
Fasting blood glucose	Ideal: <5.6 mmol/L (<100 mg/dL) untreated Intermediate: 5.6–6.9 mmol/L (100–125 mg/dL) or treated to goal Poor: ⩾7.0 mmol/L (⩾126 mg/dL)

a Ideal diet was classified using five dietary components and ideal intakes (scaled to an energy intake of 2000 kcal) and was defined as follows (a) fruits and vegetables: ⩾4.5 times per day; (b) fish: ⩾2 times per week; (c) whole grains: ⩾85 grams per day (corresponding to three 1-oz (28.3-gram) servings of fibre-rich whole grains; (d) sodium <1500 mg per day; (e) sugar-sweetened beverages: ⩽3 times per week.

US: United States standard; SBP: systolic blood pressure; DBP: diastolic blood pressure.

During the visit to the test centre, data on the iCVH components were collected [7]. Briefly, tobacco smoking was evaluated using a questionnaire asking about current and former cigarette smoking habits, age when starting smoking and years of smoking. Height and weight were assessed using standardised procedures by trained health-care staff, and BMI was calculated as weight in kilograms divided by height in metres squared (kg/m^2^). Dietary intakes were measured using a web-based food frequency questionnaire (MiniMeal-Q) which has been validated previously [9,10]. Physical activity was assessed by tri-axial accelerometer Actigraph GT3X+ (3% of participants), wGT3X+ (15% of participants) and wGT3X-BT (82% of participants; ActiGraph LCC, Pensacola, FL). The participants were instructed to use the accelerometers for seven days in an elastic belt over the right hip during all waking hours, except during water-based activities. Detailed information regarding collection and data processing has been published elsewhere [11]. For the health factors, we utilised self-reported data of diagnosis of hypertension, diabetes and hyperlipidaemia as well as objective data (from the Swedish Prescribed Drug Register) regarding medications for these conditions until one year before the measurement. Total cholesterol and blood glucose were measured using blood samples after an overnight fast at the site-specific university hospital laboratory. Venous plasma glucose was analysed through an enzymatic method with hexokinase. From 2703 participants without venous plasma glucose data, we utilised data from capillary blood samples after converting the results to plasma capillary glucose using the IFCC standard factor conversion [12]. Systolic and diastolic blood pressures were measured twice in each arm with an automatic device (Omron M10-IT; Omron Health Care Co., Kyoto, Japan). The average pressure in the arm with the highest mean blood pressure was used in the analysis.

Statistical analysis included descriptive analysis of the components of iCVH both for the overall population and stratified by sex. Sex differences in iCVH were analysed using the chi-square test. A *p*-value of <0.05 was considered statistically significant, and all tests were two sided. Analyses were performed using IBM SPSS Statistics for Windows v28 (IBM Corp., Armonk, NY).

## Results

**Figure 1.**
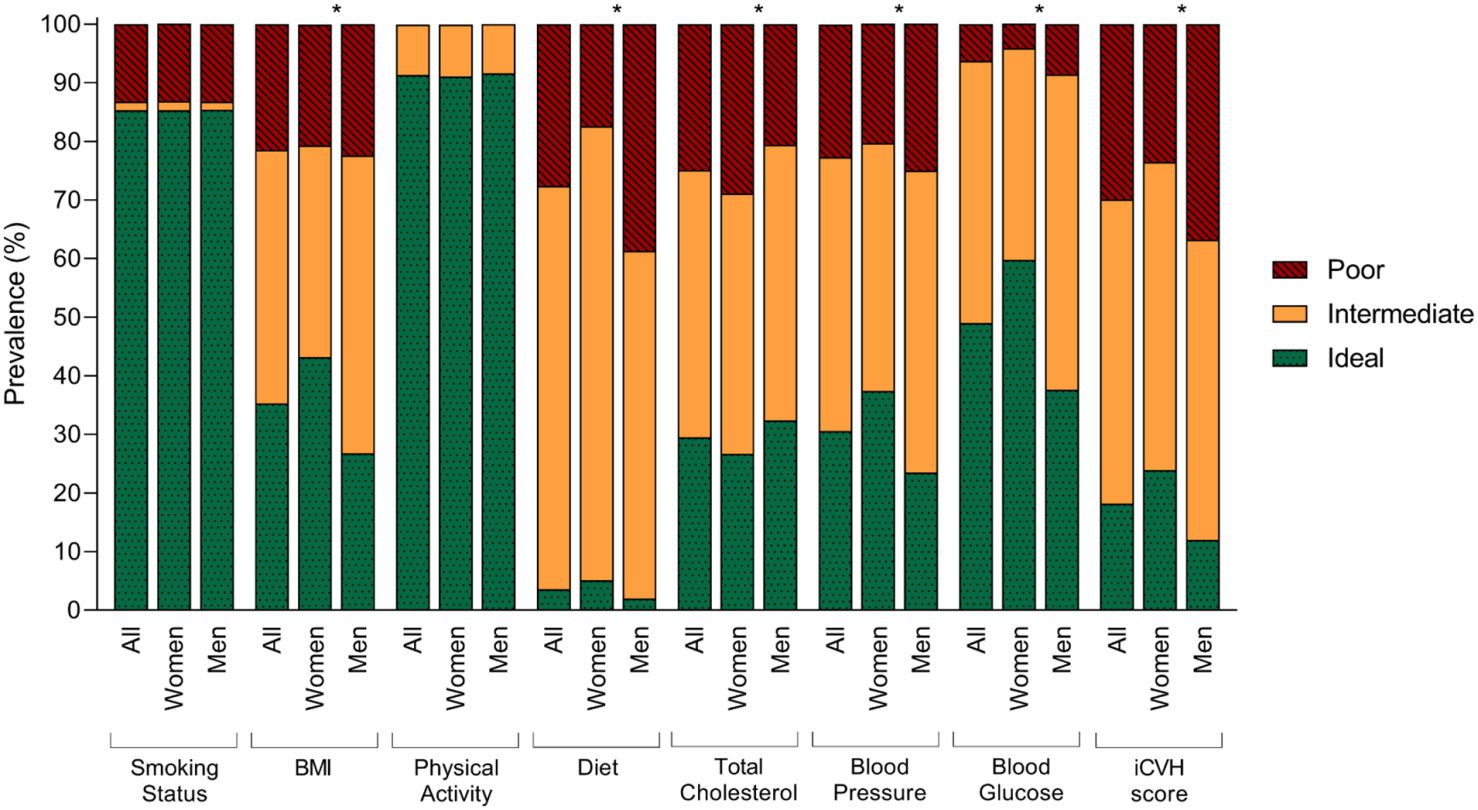
Prevalence of ideal, intermediate and poor cardiovascular health metrics, overall and stratified by sex. *p<0.05. BMI: body mass index; iCVH: ideal cardiovascular health.

Of the 30,154 participants, 51.4% (*n*=15,508) were women, and 48.6% (*n*=14,646) were men. The average age was 57.5 (*SD*=4.3) years. Figure 1 shows the prevalence of ideal, intermediate and poor cardiovascular health for the seven iCVH score metrics and the total iCVH score (detailed data in Supplemental Table SI). For the total iCVH score, we observed that only 18.2% of the population reached ideal status (i.e. ⩾5 components at the ideal level), whereas 51.9% were classified as having an intermediate status and 29.9% as having a poor status of iCVH. Women had a higher prevalence of ideal iCVH status (23.9% vs. 12.0%) and a lower prevalence of poor iCVH status (23.5% vs. 36.8%). The most prevalent individual components at ideal levels were smoking status (85.3%) and physical activity (91.3%). However, if a stricter criterion for moderate-to-vigorous physical activity (⩾300 minute per week) was applied as in a previous SCAPIS publication [13], 63.3% of the participants would be considered to have ideal physical activity. For the remaining five iCVH components, less than half of the population met the criteria for ideal levels: blood glucose (49.0%), BMI (35.3%), blood pressure (30.6%), total cholesterol (29.5%) and diet (3.6%). The estimates did not materially differ when analysing those with complete data on each component (*n*=25,993).

## Conclusions

In summary, the prevalence of iCVH was low (18.2%) in this large population-based study of Swedish middle-aged individuals, and men had in general lower iCVH than women. These findings are in alignment with previous studies of iCVH in adult populations in other parts of the world [6,14]. Our data may serve as benchmarks for future national and international comparisons and motivate efforts to promote cardiovascular health in the general population, given the strong link between iCVH with all-cause and CVD mortality and morbidity [4,5].

## Supplemental Material

sj-docx-1-sjp-10.1177_14034948221147093 – Supplemental material for Low prevalence of ideal cardiovascular health in the general Swedish population: Results from the Swedish CArdioPulmonary bioImage Study (SCAPIS) Click here for additional data file. Supplemental material, sj-docx-1-sjp-10.1177_14034948221147093 for Low prevalence of ideal cardiovascular health in the general Swedish population: Results from the Swedish CArdioPulmonary bioImage Study (SCAPIS) by Sara Higueras-Fresnillo, Ángel Herraiz-Adillo, Viktor H. Ahlqvist, Daniel Berglind, Cecilia Lenander, Bledar Daka, Maria Brännholm Syrjälä, Johan Sundström, Carl Johan Östgren, Karin Rådholm and Pontus Henriksson in Scandinavian Journal of Public Health
