# Supplementary material for: Low prevalence of ideal cardiovascular health in the general Swedish population: Results from the Swedish CArdioPulmonary bioImage Study (SCAPIS)
Source: Scand J Public Health. 2023 Jan 16;51(4):527–30. doi: 10.1177/14034948221147093 (PMC10265283; doi:10.1177/14034948221147093)
Supplement: sj-docx-1-sjp-10.1177_14034948221147093 – Supplemental material for Low prevalence of ideal cardiovascular health in the general Swedish population: Results from the Swedish CArdioPulmonary bioImage Study (SCAPIS) [file sj-docx-1-sjp-10.1177_14034948221147093.docx]

**Supplementary material**

**Low prevalence of ideal cardiovascular health in the general Swedish population: Results from the Swedish CArdioPulmonary bioImage Study (SCAPIS)**

Sara Higueras-Fresnillo^1^, Ángel Herraiz-Adillo^1^, Viktor H. Ahlqvist^2^,
Daniel Berglind^2,3^, Cecilia Lenander^4^, Bledar Daka^5^, Maria Brännholm Syrjälä^6^,
Johan Sundström^7,8^, Carl Johan Östgren^1,9^, Karin Rådholm^1,8^, Pontus Henriksson^1^

^1^ Department of Health, Medicine and Caring Sciences, Linköping University, Linköping, Sweden. [sara.higueras-fresnillo@liu.se](mailto:sara.higueras-fresnillo@liu.se)

^2^ Department of Global Public Health, Karolinska Institutet, Stockholm, Sweden.

^3^ Centre for Epidemiology and Community Medicine, Region Stockholm, Stockholm, Sweden.

^4^ Department for Clinical Sciences in Malmö, Centre for Primary Health Care Research, Lund University, Lund, Sweden.

^5^ School of public health and community medicine, Sahlgrenska Academy, University of Gothenburg.

^6^ Department of Public Health and Clinical Medicine, Family Medicine, Umeå University, Sweden.

^7^ Department of Medical Sciences, Uppsala University, Uppsala, Sweden.

^8^ The George Institute for Global Health, University of New South Wales, Sydney, Australia.

^9^ Centre for Medical Image Science and Visualization (CMIV), Linköping university, Linköping, Sweden.

**Table S1.** Prevalence of ideal, intermediate, and poor cardiovascular health in SCAPIS (n=30 154).

| **Metrics** | **Prevalence (%)** | | | **diff. by sex *p*-value** |
| --- | --- | --- | --- | --- |
|  | **Ideal** | **Intermediate** | **Poor** |  |
| ***Smoking status****,* *n=29 017* | | |  |  |
| All | 85.3 | 1.5 | 13.2 |  |
| Women | 85.3 | 1.6 | 13.2 | 0.426 |
| Men | 85.4 | 1.4 | 13.2 |  |
| ***BMI****, n=30 150* | | |  |  |
| All | 35.3 | 43.2 | 21.5 |  |
| Women | 43.2 | 36.1 | 20.6 | <0.001 |
| Men | 26.8 | 50.8 | 22.4 |  |
| ***Physical activity****, n=28 975* | | |  |  |
| All | 91.3 | 8.6 | 0 |  |
| Women | 91.1 | 8.8 | 0 | 0.251 |
| Men | 91.6 | 8.4 | 0 |  |
| ***Diet****, n=28 175* | |  |  |  |
| All | 3.6 | 68.8 | 27.6 |  |
| Women | 5.1 | 77.5 | 17.4 | <0.001 |
| Men | 2.0 | 59.3 | 38.7 |  |
| ***Total cholesterol****, n=30 062* | | |  |  |
| All | 29.5 | 45.6 | 24.9 |  |
| Women | 26.7 | 44.4 | 28.9 | <0.001 |
| Men | 32.4 | 47.0 | 20.6 |  |
| ***Blood pressure****, n=29 995* | | |  |  |
| All | 30.6 | 46.7 | 22.6 |  |
| Women | 37.4 | 42.3 | 20.4 | <0.001 |
| Men | 23.5 | 51.5 | 25.1 |  |
| ***Blood Glucose****, n=29 765* | | |  |  |
| All | 49.0 | 44.7 | 6.3 |  |
| Women | 59.8 | 36.1 | 4.2 | <0.001 |
| Men | 37.6 | 53.8 | 8.6 |  |
| ***Total iCVH score***^a^, *n =25 993* | | |  |  |
| All | 18.2 | 51.9 | 29.9 |  |
| Women | 23.9 | 52.6 | 23.5 | <0.001 |
| Men | 12.0 | 51.2 | 36.8 |  |

iCVH, ideal cardiovascular health. ^a^ Classified as ideal (≥5 ideal components), intermediate (3-4 ideal components), and poor (≤2 ideal components)
